# APOE Effects on Default Mode Network in Chinese Cognitive Normal Elderly: Relationship with Clinical Cognitive Performance

**DOI:** 10.1371/journal.pone.0133179

**Published:** 2015-07-15

**Authors:** Haiqing Song, Haixia Long, Xiumei Zuo, Chunshui Yu, Bing Liu, Zhiqun Wang, Qi Wang, Fen Wang, Ying Han, Jianping Jia

**Affiliations:** 1 Department of Neurology, Xuanwu Hospital Capital Medical University, Beijing, China; 2 Institute of Automation, Chinese Academy of Sciences, Beijing, China; 3 Department of Radiology, Xuanwu Hospital Capital Medical University, Beijing, China; Banner Alzheimer's Institute, UNITED STATES

## Abstract

**Background:**

Functional connectivity in default mode network (DMN) may be changed in Alzheimer’s disease (AD) patients and related risk populations, such as amnestic mild cognitive impairment (aMCI) patients and APOE ε4 carriers. Exploring DMN changes and related behavioral performance of APOE ε4 population might provide valuable evidence for better understanding the development of AD.

**Methods:**

Subjects were enrolled from a population-based cohort established in a multi-center study in China. Forty-nine cognitive normal individuals were enrolled after standardized cognitive evaluations, MRI examination and APOE genotyping. Regions of interest (ROI)-based functional connectivity analyses were performed, and voxel-based analyses were used to validate these findings. The correlation between DMN functional connectivity and behavioral performance was further evaluated between APOE ε4ε3 and ε3ε3 carriers.

**Results:**

Comparing to ε3ε3 carriers, functional connectivity between left parahippocampal gyrus and right superior frontal cortex (LPHC-R.Sup.F), left parahippocampal gyrus and medial prefrontal cortex (ventral) (LPHC-vMPFC) were significantly increased in ε4ε3 carriers, while connectivity between cerebellar tonsils and retrosplenial was decreased. LPHC-R.Sup.F connectivity was positively correlated with the changes of delay recall from baseline to follow-up (r = 0.768, p = 0.009), while LPHC-vMPFC connectivity had a positive correlation with MMSE at baseline (r = 0.356, p = 0.018), and a negative correlation with long-delayed recognition at follow-up (r = -0.677, p = 0.031). Significantly increased functional connectivity in vMPFC was confirmed in voxel-based analyses by taking LPHC as seed region.

**Conclusion:**

APOE ε4 carriers present both increased and decreased functional connectivity in DMN, which is correlated with clinical cognitive performances. DMN changes might be an early sign for cognitive decline.

## Introduction

Alzheimer’s disease (AD) is the most common neurodegenerative disease in elderly, but effective treatments are limited. Therefore, researches are trying to understand the development of AD, and look for early sign of cognitive decline which might be treatable at early stage.

Neuroimaging studies is an useful tool to evaluate structural, functional, and metabolic changes of the brain, and widely used in investigation of AD and associated diseases. Default mode network (DMN) is one of the highlighted network, which consists of a collection of brain structures including the medical prefrontal cortex (mPFC), posterior cingulate cortex (PCC), precuneus, anterior cingulate cortex (ACC), parietal cortex, and hippocampus[1]. It presents increased connectivity during rest and decreased connectivity during specific goal directed behaviors[2].

DMN is considered to be associated with AD, since the regions involved are core structures for memory system, and vulnerable to deposition of amyloid protein[3,4]. Moreover, there are numerous evidence of disrupted functional connectivity (FC) of DMN in AD patients[5,6] and related risk populations, such as amnestic mild cognitive impairment (aMCI) patients[7,8]and APOE ε4 carriers[9–12]. Besides, some studies showed increased FC in AD and related diseases, which has been explained by compensatory-recruitment hypothesis[13].

APOE ε4 allele has been associated with an increased risk for AD[14], and therefore the APOE ε4 carriers are considered to be good candidates for learning the development of AD. Similar imaging studies on DMN have been carried out in APOE4 carriers. Both decreased and increased functional connectivity were observed within different regions in the DMN in older asymptomatic APOE4 carriers[9–12]; while increased functional connectivity was also found in younger APOE4 carriers[15,16], which suggested a compensatory mechanism to maintain normal cognitive performances decades before clinical manifestation.

However, the clinical meanings of these functional connectivity have not been fully investigated, and relevant literatures are limited. Westlye and colleagues found significant negative correlation between memory performance and resting hippocampal connectivity synchronization in older cognitive normal individuals using the Norwegian translation of the California Verbal Learning Test, second version (CVLT-II), suggesting a potential relationship between functional connectivity and clinical cognition[10]. Therefore, It is important to confirm these findings, which would be very useful for better understanding the associations between imaging findings and clinical manifestations.

To validate the significant relationships between DMN functional connectivity changes and clinical cognitive performances, we presented here an imaging study in cognitive normal elderly from a population with two visits of cognitive measurements in Beijing, China, to investigate the APOE ε4 effects on DMN connectivity in Chinese, and further evaluate the associations between the changed functional connectivity and cognitive performances.

## Materials and Methods

### Subjects

Cognitive normal elderly were enrolled from a population-based cohort established by a multi-center study in China. This cohort was set up in 2009, aiming to screen MCI and AD patients in a well-defined population. Totally 10,276 individuals aged over 65 years were enrolled in this cohort. Cognitive measurements were evaluated at baseline, and blood samples were collected. Follow-ups were scheduled every year. Individuals from Beijing with: (a) Clinical Dementia Rating (CDR) score of 0; (b) no neurological or psychiatric disorders; (c) normal activities described in a daily living scale were invited and voluntary to participant in this study at baseline, of which 161 participants took part in the MRI examination. All scans were reviewed by neuroimaging experts. Individual with brain tumors, recent infarctions, or global abnormal signals were excluded. 99 individuals were defined as normal after cognitive evaluations at baseline, of which 64 subjects has APOE genotype information. 14 (21.9%) subjects were excluded because they were ε2 carriers, which is suggested to be a protective factor of cognition (ε3ε2 n = 11; ε4ε2 n = 3). One more subjects (ε3ε3) was excluded due to incompletion of fMRI data. Therefore, our final study population comprised of 49 cognitive normal individuals, including 14 ε4ε3 carriers and 35 ε3ε3 carriers.

### Ethics

The study was approved by the Ethics Committee of Xuanwu Hospital Capital Medical University. All subjects were informed the purpose of this study, and informed consent was signed by each subject or their legal guardian.

### Cognitive measurements

Standardized general and neurologic examination were performed on each participant. Neuropsychological tests were performed by neurologists, including mini-mental state examination (MMSE), Montreal cognitive assessment (MoCA), World Health Organization-University of California-Los Angeles auditory verbal learning test (WHO-UCLA AVLT), center for epidemiologic studies-depression (CESD), and Hachinski ischemic index (HIS). The clinical dementia rating scale (CDR) was also administered and detailed cognitive impairment history was inquired. Subjects with CDR scored as”0” and without any complaint of cognitive decline were considered to be normal.

### Genotyping

Peripheral blood samples were collected at baseline of the cohort. Genomic DNA was extracted via a modified phenol/chloroform extraction procedure. APOE genotype was determined by one-stage polymerase chain reaction (PCR) as suggested by Wenham et al [17]. Distributions of APOE genotypes were in Hardy-Weinberg Equilibrium in all subjects, and the frequency of ε4 carriers was in accordance with the known frequency of this allele in Chinese population. Observed genotypic frequencies were as follows: ε3ε2 17.2%, ε4ε2 4.7%, ε4ε3 21.9%,ε3ε3 54.7%.

### MRI data acquisition and preprocessing

MRI data was obtained using a 3.0-Tesla MR scanner (Magnetom Trio, Siemens, Erlangen, Germany). Functional images were collected axially using an echo-planar imaging (EPI) sequence. During the resting-state scanning, subjects were instructed to keep still with their eyes closed. The parameters were as follows: slices = 32, TR = 2000 ms, TE = 30 ms, thickness = 3.0 mm (with 1mm gap), FOV = 220×220 mm, matrix = 64×64, flip angle (FA) = 90°. For each subject, the fMRI scan during the resting-state provided 270 volumes. 3D T1-weighted magnetization-prepared rapid gradient echo (MPRAGE) sagittal images were collected by using the following parameters: TR = 1900 ms, TE = 2.2 ms, inversion time (TI) = 900 ms, FA = 9°, resolution = 256 × 256 matrix, slices = 176, thickness = 1.0 mm.

We preprocessed the fMRI data with the following steps using SPM5 (http://www.fil.ion.ucl.ac.uk/spm/software/spm5/) and in-house software: (1) slice timing; (2) realigning the volumes to the first volume; (3) spatially normalizing to a standard EPI template and making a resample to 2 mm *2 mm *2 mm; (4) spatially smoothing; (5) performing linear regression to remove the influence of head motion, whole brain signals and linear trends; (6) temporal band-pass filtration (0.01–0.08Hz). The parameters obtained from movement correction suggested that the maximum displacement in each cardinal direction (x, y, z) was less than 2 mm, and the maximum spin (x, y, z) was less than 2° for each participant.

### ROI-based individual DMN functional connectivity

We used the same regions of interest (ROI) to define the default network and performed the similar default network functional connectivity analyses here as reported in our previous studies[18,19]. The detailed coordinates of these 13 ROIs are shown in Table 1. All ROIs were defined as a spherical region with a radius of 6 mm at the center of the obtained coordinates for a specific ROI. We extracted the averaged BOLD time series separately for 13 ROIs in each subject, and then calculated the Pearson’s correlation coefficient between the any two averaged time series. We then transformed the resulting correlation into Z score by r-to-z transformation. Finally, we got a 13*13 DMN functional connectivity graphs for each subject.

**Table 1:** Thirteen Regions of Interest in DMN.

Brain region	Abbreviations	MNI Coordinates
Medial prefrontal cortex (anterior)	aMPFC	(-3,54,18)
Left superior frontal cortex	L.Sup.F	(-15,54,42)
Right superior frontal cortex	R.Sup.F	(18,42,48)
Medial prefrontal cortex (ventral)	vMPFC	(-6,36,-9)
Left inferior temporal cortex	L.IT	(-60,-9,-24)
Right inferior temporal cortex	R.IT	(57,0,-27)
Left parahippocampal gyrus	L.PHC	(-24,-18,-27)
Right parahippocampal gyrus	R.PHC	(27,-18,-27)
Posterior cingulated cortex	PCC	(-3,-48,30)
Retrosplenial	Rsp	(9,-54,12)
Left lateral parietal cortex	L.LatP	(-48,-69,39)
Right lateral parietal cortex	R.LatP	(48,-66,36)
Cerebellar tonsils	Cereb	(-6,-54,-48)

### Parahippocampal functional connectivity analyses

To validate the ROI-based DMN findings, we extracted left parahippocampal gyrus, which was suggested to be involved in the changed functional connectivity in preliminary analysis, as a seed region, and then performed voxel-based whole brain functional connectivity analyses. We computed the whole brain functional connectivity by calculating the Pearson correlation coefficients between the averaged time series of left parahippocampal gyrus and each voxel in the whole brain for each individual.

### Statistical analysis

Demographic information and cognitive measurements were analyzed using two-sample *t-test* and *Chi-square* test. We used a two-sample *t*-test to examine differences in the strength of connectivity between the two APOE genotype groups, meanwhile, a permutation-based correction method[20] was used to correct for multiple comparisons. For each seed region in whole brain analyses, we performed a two-sample *t*-test to examine the significance of the differences in the voxel-based default network between individuals with two different genotypes. The Pearson correlation was calculated to evaluate the correlation between DMN functional connectivity and behavioral performance.

## Results

### Demographic information and cognitive measurements

Totally 49 individuals were enrolled in our study, of whom 14 individuals were ε4 carriers (aged 75.93±5.28y, Male 42.9%), while 35 individuals were ε3ε3 carriers (aged 72.11±4.09y, Male 37.1%). 47 subjects (34 ε3ε3 and 13 ε4ε3 carriers) at baseline and 12 subjects (11 ε3ε3 and 2 ε4ε3 carriers) at follow-up with completed cognitive measurements were included in further analysis for associations between functional connectivity and behavior performance. One more subject (ε3ε3 carrier) was excluded from the analysis involving baseline MMSE due to the far deviation from the average score (>3SD). Average scores of Mini-Mental State Examination (MMSE), Delay recall and Long-delayed recognition were not significantly different between ε3ε3 and ε4ε3 carriers both at baseline and follow-up (Table 2).

**Table 2:** Comparison of demographic information and cognitive measurements between APOE ε3ε3 and ε4ε3 carriers.

Variable	APOE ε3ε3	APOE ε4ε3	P
***Demographic*, *N***	35	14	
Age	72.11±4.09	75.93±5.28	0.009
Gender, Male, %(N)	37.1 (13)	42.9 (6)	0.711
***Cognitive measurements(baseline)*,*N***	34	13	
MMSE	26.56±3.33	28.23±1.96	0.097
Delay recall	9.79±2.20	9.85±2.70	0.946
Long-delayed recognition	12.15±3.32	13.38±1.76	0.210
***Cognitive measurements(follow-up)*,*N***	11	2	
MMSE	25.73±3.41	24.50±3.54	0.650
Delay recall	8.55±2.58	5.50±0.71	0.137
Long-delayed recognition	10.64±2.34	11.50±0.71	0.625

### APOEε4 and DMN Functional connectivity

**Fig 1:**
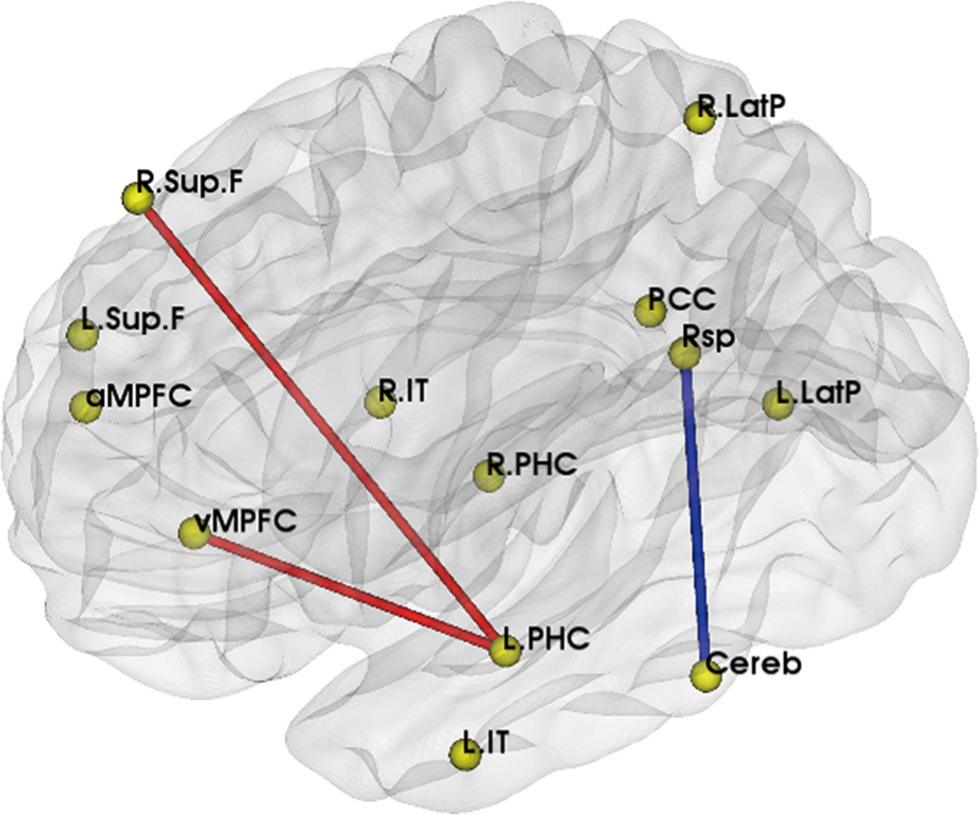
Significantly different DMN functional connectivity between APOE ε4ε3 and ε3ε3. Brain model for the changed functional connectivity: the red line represented the increased functional connectivity in ε4ε3 carriers, and blue line represented the decreased one.

We first compared ROIs functional connectivity between ε3ε3 and ε4ε3 carriers. Comparing to ε3ε3 carriers, functional connectivity between left parahippocampal gyrus and right superior frontal cortex (LPHC-R.Sup.F, p_raw_ = 0.028, p_adjusted_ = 0.026), left parahippocampal gyrus and medial prefrontal cortex (ventral) (LPHC-vMPFC, p_raw_ = 0.004, p_adjusted_ = 0.010) were significantly increased in ε4ε3 carriers, while connectivity between cerebellar tonsils and retrosplenial (Cereb-Rsp, p_raw_ = 0.030, p_adjusted_ = 0.016) was decreased, after controlling for the effects of age and gender (Fig 1).

### Functional connectivity and cognitive measurements

**Fig 2:**
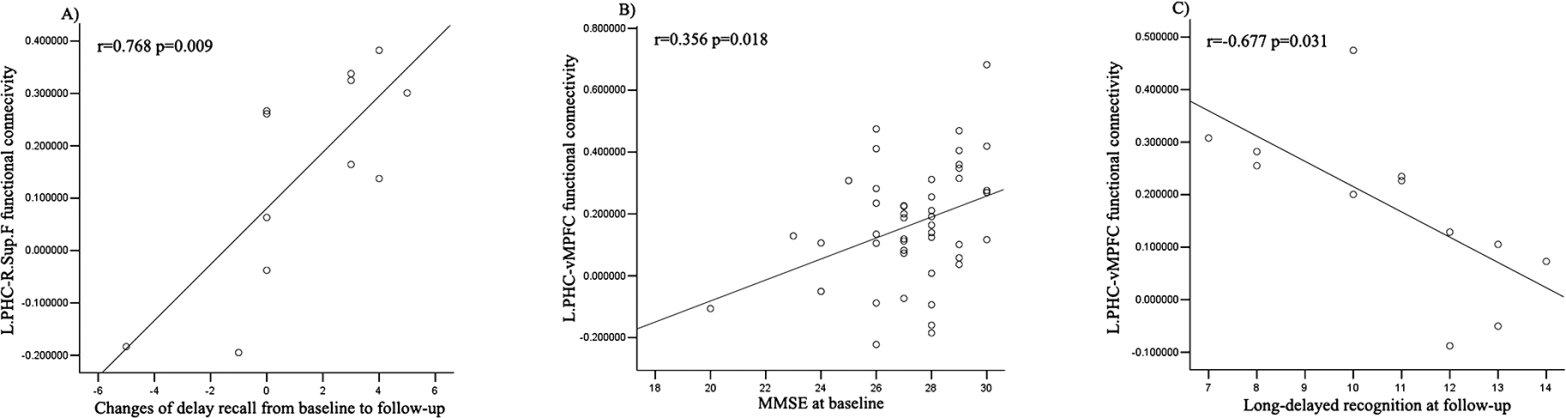
Changed functional connectivity was associated with cognitive performances. A. The increased LPHC-R.Sup.F functional connectivity was positively correlated with the changes of delay recall from baseline to follow-up. B. The increased LPHC-vMPFC functional connectivity had a positive correlation with MMSE at baseline, and C. a negative correlation with long-delayed recognition at follow-up.

To evaluate the effects on clinical manifestations from these changed functional connectivity, we further analyzed the association between each connection and neuropsychological tests (Fig 2). After controlling for age and gender, we found that the LPHC-R.Sup.F functional connectivity was positively correlated with changes of delay recall from baseline to follow-up (r = 0.768, p = 0.009). For LPHC-vMPFC functional connectivity, we found that it had a positive correlation with MMSE at baseline (r = 0.356, p = 0.018), and a negative correlation with long-delayed recognition at follow-up (r = -0.677, p = 0.031).

### Voxel-based LPHC functional connectivity analyses

**Fig 3:**
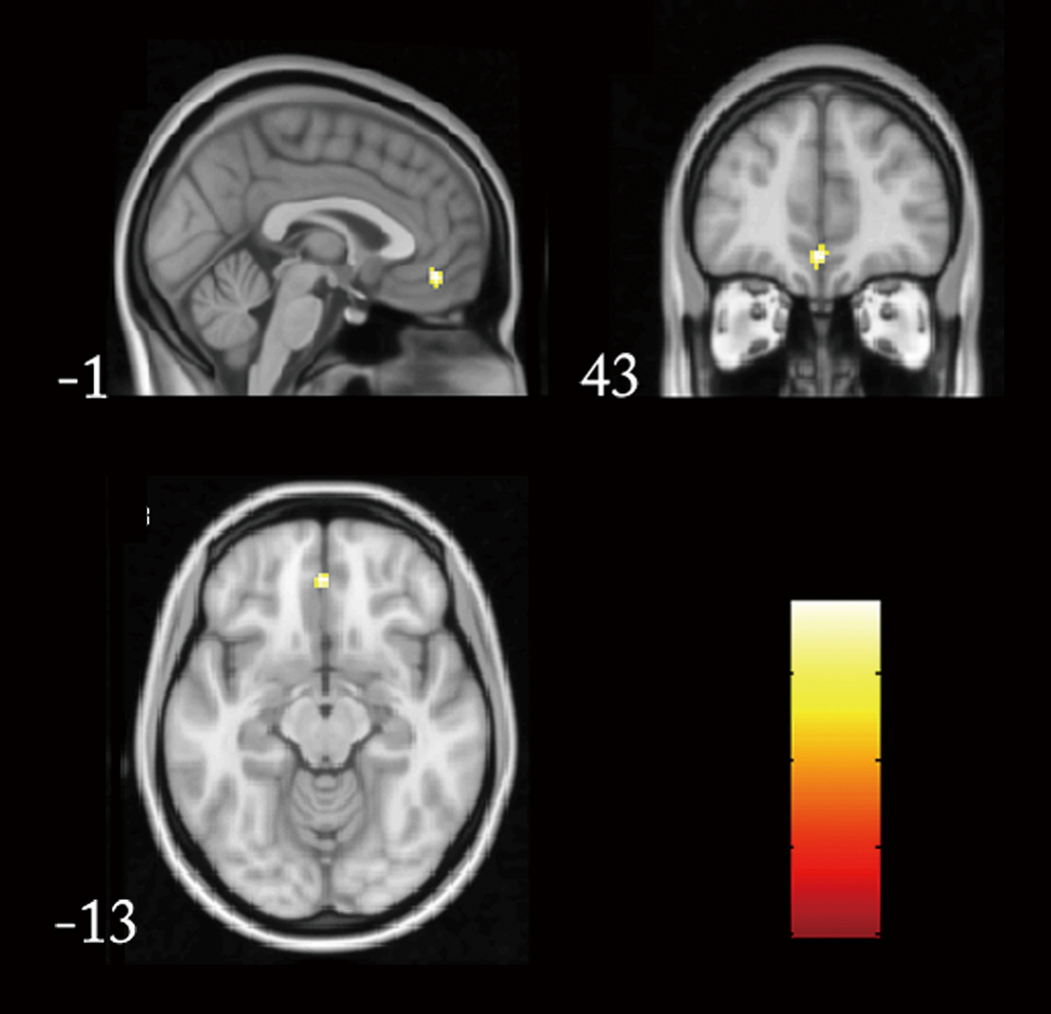
Whole brain functional connectivity by taking LPHC as a seed region. Significantly increased functional connectivity presented in medial prefrontal cortex (cluster size = 20, p = 0.005) in APOE ε4 carriers were presented from different directions.

To verify our ROI-based findings, we also performed the voxel-based whole brain functional connectivity analyses by taking LPHC as a seed region. When the individuals’ functional connectivity was calculated and compared between APOE ε4 carriers and ε3ε3 genotypes, we found significantly increased functional connectivity in medial prefrontal cortex (cluster size = 20, p = 0.005, Fig 3), which partially supported the ROI-based functional connectivity findings.

## Discussion

Our study demonstrated APOE ε4 related changes in DMN in cognitive normal elderly, with decreased connectivity between cerebellar tonsils and retrosplenial, and increased LPHC-R.Sup.F and LPHC-vMPFC connectivity in ε4ε3 carriers, compared to ε3ε3 carriers. Whole brain analysis confirmed increased functional connectivity between MPFC and LPHC, which suggested specific effects of APOE on DMN. Moreover, we found the significant correlations between functional connectivity and clinical cognitive performances, indicating potential functional role of DMN.

Neuroimaging studies have identified the effects of APOE ε4 allele on both brain structure and metabolism[21–24]. APOE ε4 carriers may present gray matter reductions, decreased resting glucose metabolism in brain regions with potential AD pathology, including the posterior cingulate, parietal, temporal, and prefrontal cortices, and also increased task-related activation in relative regions. Recently, altered DMN connectivity has been reported in APOE ε4 carriers. Westlye and colleagues reported increased functional connectivity between hippocampus and the posterior DMN in APOE4 carriers [10], and their whole-brain analysis revealed similar effects in the PCC, parietal, and parahippocampal regions. These findings remained significant even hippocampal volumes and gray matter maps were considered as covariates. Another case-control study used seed-based voxel-wise connectivity analysis, and found increased connectivity in the cingulate gyrus, medial prefrontal cortex, bilateral insular cortex, striatum, and thalamus in ε4 carriers when ACC was considered as a seed region[12]. They also found decreased connectivity between PCC and the posterior DMN in APOE4 carriers. Fleisher et al reported increased DMN connectivity in the medial and dorsolateral prefrontal cortex and temporal lobe structures in cognitively normal APOE4 carriers, with posterior cingulate/retrospenial region (pC/rsp) as a seed region[9]. Sheline et al presented a study in 2010 using bilateral precuneus as a seed region, and suggested that most of DMN regions, particularly bilateral hippocampus and left parahippocampus, had decreased connectivity with precuneus in carriers[25]. Not only in the old population, similar findings were presented in young APOE4 carriers. Filippini et al found increased DMN coactivation (including retrosplenial, medial temporal, and medial-prefrontal cortical areas) in APOE4 young carriers (20–35 years)[15]. Later, Dennis and colleagues replicated this findings[16]. This suggested a long-term effect of APOE4 on DMN connectivity, even decades before the onset of AD. Our results were consistent with these studies though there was some differences in regions involved.

The decreased functional connectivity in DMN has been considered to be attributed to the early pathology of AD in APOE4 carriers, which is in line with the disruption of white matter tracts and beta amyloid deposition in DMN reported recently[26,27]. Amyloid-β plaques are an important early event in the pathogenesis of AD. Since the APOE ε4 is the risk allele for AD, we speculate that the group APOE ε3ε4 allele might have more amyloid-β deposition, which result in the myelin breakdown, and in turn disrupt functional connectivity. On the other hand, the increased functional connectivity has been interpreted as a compensation for normal cognitive performances. When the functional connectivity between some regions showed decrease in the group APOE ε3ε4, the balance was disrupted, the subjects commonly recruit more other regions and strengthen the connectivity to counteract the neurobiological changes due to APOE ε4.

Neurocognitive significances of these functional connectivity are still under investigation. Fleisher et al found a positive trend association between CVLT scores and DMN connectivity z-scores in the precuneus in APOE4 carriers[9], while anther study reported a negative correlation between DMN synchronization and memory performance[10]. we found a positive correlation between increased LPHC-vMPFC functional connectivity and baseline MMSE scores in this study, which proved the impact of changed functional connectivity on clinical cognitive manifestations. Moreover, the negative correlation between increased functional connectivity and cognitive performance at follow-up supported the hypothesis that these increased functional connectivity is a compensative mechanism to maintain normal cognition, but with changes over time, this compensation might not be enough for perseveration of cognitive performance, and decline of cognition would be presented.

Currently, changes of DMN are identified not only in AD, but also in AD risk population, such as APOE4 carriers and MCI patients. This suggests its potential value for early prediction and diagnosis of AD. Sheline and colleagues performed a fMRI study in individuals without preclinical fibrillar amyloid deposition (Pittsburgh Compound B, PIB−)[25] and found that most regions associated with decreased connectivity in whole brain analysis were located in DMN, suggesting early damage of DMN even before pathological manifestation. In addition, Koch et al. investigated diagnostic power of DMN in the detection of AD using both ROI-based signal time course evaluations and independent component analyses (ICA)[28]. Multivariate model combining both the activity of various parts in DMN and also the interconnectivity between these regions was proved to enhance the diagnostic power. Moreover, a model developed for AD identified an AD typical pattern in 11 of 17 MCI patients, with a similar percentage of MCI subjects presenting an AD typical pattern in a recent PET study (79% in subjects with impairment in multiple cognitive domains and 31% in aMCI patients)[29]. These findings suggested that DMN might be a good candidate to be biomarkers of AD prediction and early diagnosis. Changes of DMN connectivity were found to be associated with progress of clinical cognitive performance in our study, which also supported its potential predicting role in cognitive decline.

Different analytical approaches could influence the results for DMN connectivity. Here we used ROI-based analysis with the ROI that has been proved in our previous studies to define DMN[18,19], which might minimize the impact of inaccurate ROIs. Furthermore, we used seed-based correlation analysis to verify the findings from ROI-based analysis. Though the age of two groups were not perfectly matched, our subjects were enrolled from a population-based cohort, which might be more representative. Moreover, we used statistical methods to control the effects of age and gender. It is a pity that there were several missing data in clinical cognitive measurements, therefore the sample size for related analysis was limited, especially in follow-ups. Further studies should be carried out in larger population with completed clinical information to verify the findings between changed functional connectivity and neurocognitive performance.

Another analytic issue is multiple comparison for ROI-based analyses in our study. Here we used a permutation-based correction to assess the significance of the P (t-test) value for any given connectivity. The permutation test is similar to the Bonferroni correction in that it controls the probability of finding one connectivity by chance in the hypotheses tested; however, a stringent Bonferroni correction is known to be extremely conservative and can thus lead to unacceptable levels of Type II (i.e. false negative) errors in multiple testing; especially when the test statistics are highly interdependent. Meanwhile, a permutation-based correction is data-dependent and has been widely accepted and recommended in studies that involved multiple statistical testing[20,30,31]. Under the threshold at raw P = 0.05 or adjusted P = 0.05, we can get the same 3 significantly different connectivity within the default network.

## Conclusion

Our study suggested significant effects of APOE4 on functional connectivity in DMN in older Chinese population, which might have important correlations with clinical cognition. Clinicians might need to consider the genetic influence on DMN and cognitive performance in clinical practice.
